# Intraocular lens power calculation in eyes with previous corneal refractive surgery

**DOI:** 10.1186/s40662-018-0110-5

**Published:** 2018-07-08

**Authors:** Giacomo Savini, Kenneth J. Hoffer

**Affiliations:** 1 G.B. Bietti Eye Foundation, Rome, Italy; 2 Stein Eye Institute, University of California, Los Angeles, CA USA; 3 St. Mary’s Eye Center, Santa Monica, CA USA

**Keywords:** Cataract, Intraocular lens power, LASIK, PRK, Excimer laser, Corneal surgery, Keratometry

## Abstract

**Background:**

This review aims to explain the reasons why intraocular lens (IOL) power calculation is challenging in eyes with previous corneal refractive surgery and what solutions are currently available to obtain more accurate results.

**Review:**

After IOL implantation in eyes with previous LASIK, PRK or RK, a refractive surprise can occur because i) the altered ratio between the anterior and posterior corneal surface makes the keratometric index invalid; ii) the corneal curvature radius is measured out of the optical zone; and iii) the effective lens position is erroneously predicted if such a prediction is based on the post-refractive surgery corneal curvature. Different methods are currently available to obtain the best refractive outcomes in these eyes, even when the perioperative data (i.e. preoperative corneal power and surgically induced refractive change) are not known. In this review, we describe the most accurate methods based on our clinical studies.

**Conclusions:**

IOL power calculation after myopic corneal refractive surgery can be calculated with a variety of methods that lead to relatively accurate outcomes, with 60 to 70% of eyes showing a prediction error within 0.50 diopters.

## Background

Calculating the intraocular lens (IOL) power in eyes with prior corneal refractive surgery is still a challenging task for all ophthalmologists. IOL power calculation is not a perfect science even in unoperated eyes, where 20-25% of cases can suffer from a prediction error in refraction higher than 0.5 diopters (D), notwithstanding modern formulas and instruments. Such a percentage is likely to increase when the cornea has been treated by any kind of refractive surgery, especially if standard calculations are performed. The effort of several investigators during the last 15 years lead to the publication of more than 30 methods to overcome this problem and the most recent studies have shown that good refractive outcomes can be achieved if the appropriate methods are selected. This review aims 1) to explain why IOL power calculation after corneal refractive surgery is difficult and 2) to suggest the methods that have been shown to be the most accurate ones.

## Review

### Why is intraocular lens (IOL) power calculation so difficult after corneal refractive surgery?

#### Excimer laser surgery

In eyes with prior photorefractive keratectomy (PRK) or laser in situ keratomileusis (LASIK), IOL power can be erroneously calculated for three reasons. Firstly, any keratometer and corneal topography system is unable to calculate the diopters to be entered into IOL power calculation formulas as a measure of the central corneal power. This is known as the “keratometric index error” [1, 2]. The reason for this difficulty lies in the fact that these instruments use a standardized, fictitious keratometric index of refraction (usually 1.3375) to convert the measured radius of the anterior corneal surface into keratometric diopters, on the basis of the paraxial equation:

$$ \mathrm{P}=\left(\mathrm{n}-1\right)/\mathrm{r} $$

where P is the corneal power (in D), n is the keratometric index of refraction and r is the radius of curvature of the anterior corneal surface (in meters). The value of 1.3375 dates back to the nineteenth century and was formulated so that a corneal radius of 7.5 mm corresponds to a corneal power of 45 D [3].

While keratometers and topographers measure the radius of the anterior corneal curvature, the keratometric index of refraction refers to a theoretical single refractive lens representing both corneal surfaces. It assumes a constant ratio of anterior to posterior corneal curvature. Such an assumption works well in virgin eyes, but, when the anterior corneal curvature is altered by corneal refractive surgery (and the posterior curvature does not change) such a ratio is disrupted and the usual keratometric refractive index becomes invalid.

As a consequence, after myopic correction, keratometry readings usually overestimate corneal power and the resulting IOL power is underestimated, so that patients are likely to experience postoperative hyperopia [4–8]. Conversely, in the event of hyperopic correction, corneal power is underestimated, IOL power is overestimated and patients risk postoperative myopia [9]. Usually, the higher the attempted correction, the higher the resulting under or over/under correction.

It has been shown that, after myopic excimer laser surgery, the keratometric index of refraction changes should be decreased proportionally to the amount of correction in order to get correct measurements of corneal power [10–12]. Alternatively, the keratometric index error can be overcome by measuring the curvature of both corneal surfaces by technologies, such as Scheimpflug imaging or optical coherence tomography. The latter approach, however, requires specifically optimized formula constants.

A second problem, known as the “radius error” or “instrument error” [1, 2], is related to another assumption made by most devices, which extrapolate the central corneal curvature from paracentral measurements. After myopic corneal ablations, these instruments can measure a steeper corneal curvature than in the central area. Accordingly, different authors have shown that central corneal curvature measurements better reflect the refractive change induced by surgery [13, 14], and have suggested using the central values provided by corneal topography, rather than simulated keratometry (SimK), to calculate the corneal power after excimer laser surgery [15–17]. This issue is clinically relevant in cases of small or decentered treatments, where the corneal radius may be measured on the periphery of the treated zone and be different with respect to that passing through the visual axis. Otherwise, when the optical zone is equal or larger than 6 mm the radius error is negligible [18].

Thirdly, third-generation IOL power formulas (Hoffer Q, Holladay 1 and SRK/T) use corneal power to predict the effective lens position (ELP). After myopic LASIK or PRK, the low postoperative corneal power leads to an underestimation of the ELP and further contributes to IOL underestimation. The opposite effect occurs after hyperopic surgery. To solve this issue, Jaime Aramberri, MD, developed the Double-K method, which uses two K-values: the pre-refractive surgery K for the calculation of the ELP and the post-refractive surgery K for the vergence formula that finally calculates the IOL power [19]. This error does not occur with some formulas, such as the Haigis’ [20], which do not estimate the ELP from the corneal power.

In fact, a fourth error can be hypothesized, i.e. the change in corneal asphericity induced by the laser ablation. Since corneal asphericity plays a role in IOL power calculation in unoperated eyes [21], it is logical to expect that it is even more important after excimer laser, although this issue has received little attention.

#### Incisional surgery

Incisional techniques like radial keratotomy (RK) do not induce any loss of corneal tissue. For many years it has been postulated that RK produces a similar flattening of both corneal surfaces, which deform in parallel, so that the ratio between them is maintained and the keratometric index of refraction (1.3375) is still valid [2]. Scheimpflug imaging allowed us to show that after RK, the posterior corneal surface undergoes a more evident flattening than the anterior one, so that the keratometric index is no longer valid [22]. In addition, the typically small (3.0 mm) optical zones make the radius error likely. This may produce an overestimation of the corneal power, underestimation of the IOL power and hence postoperative hyperopia [23–25].

Moreover, the unpredictability of IOL power calculation after RK can worsen due to the mechanical instability of the cornea following incisional surgery. Phacoemulsification may temporarily reopen the keratorefractive incisions as if the keratorefractive procedure had just been carried out [25]. This instability may exacerbate central flattening and peripheral bulging; often it restabilizes but residual flattening may sometimes persist.

## Methods to calculate IOL power after LASIK and PRK

The increasing number of post-LASIK/PRK patients undergoing cataract surgery and the increasing refractive expectations has led the ophthalmic community to describe more than 30 methods to calculate IOL power in these eyes over the last 15 years. Such a large number of methods can improve the chances of achieving the desired outcome, but can also generate some confusion among surgeons who do not know which is the best method for their patients. Several papers have compared some of these methods [4, 7, 16, 26–34], and we are now better able to suggest the best options for each patient, depending on the availability of preoperative and postoperative data. Based on a literature search through PubMed, we selected all papers dealing with IOL power calculation after myopic refractive surgery and published a series of studies to identify the methods that yielded the most accurate outcomes [28, 31, 32]. The following is a description of these methods and their results.

### When preoperative keratometry and/or refractive change are available

#### Seitz/Speicher’s method

This method relies on preoperative keratometry and does not need information about the refractive change [3, 35]. It assumes that the total dioptric power of the cornea (P), as measured by keratometry, can be calculated by adding the power of the anterior (P_a_) and posterior (P_p_) corneal surfaces:

$$ \mathrm{P}={\mathrm{P}}_{\mathrm{a}}+{\mathrm{P}}_{\mathrm{p}}=\left({\mathrm{n}}_2-{\mathrm{n}}_1\right)/{\mathrm{r}}_1+\left({\mathrm{n}}_3-{\mathrm{n}}_2\right)/{\mathrm{r}}_2 $$

where n_1_ is the refractive index of air (= 1), n_2_ is the refractive index of the cornea (= 1.376) and n_3_ is the refractive index of the aqueous humor (= 1.336). Both preoperatively and postoperatively, the power of the anterior corneal surface (P_a_) can be obtained by multiplying the corneal power by 1.114 (corresponding to 376/337.5) [16, 32]. Hence:

$$ {\mathrm{P}}_{\mathrm{a}}=\mathrm{SimK}\times 1.114 $$

Knowing the power of the anterior corneal surface allows us to calculate, prior to surgery, the power of the posterior corneal surface (P_p_) according to the formula:

$$ {\mathrm{P}}_{\mathrm{p}}={\mathrm{P}}_{\mathrm{a}}-\mathrm{P}=\left(\mathrm{SimK}\times 1.114\right)-\mathrm{SimK} $$

After LASIK or PRK, the power of the anterior corneal surface can then be added to that of the posterior corneal surface (which is assumed to be unchanged), as expressed by the formula

$$ \mathrm{P}=\mathrm{postpop}\ {\mathrm{P}}_{\mathrm{a}}+{\mathrm{P}}_{\mathrm{p}}=\mathrm{postpop}\ \mathrm{SimK}\times 1.114+\left(\mathrm{preop}\ \mathrm{SimK}\times 1.114-\mathrm{preop}\ \mathrm{SimK}\right) $$

This method has been shown to provide excellent results when combined with the Double-K SRK/T formula [28, 31, 32]. The main advantage of this method is that it does not require perioperative refractive data, as the preoperative K readings are sufficient.

If the preoperative corneal power is unavailable, this method can still be adopted using the modification by Savini, i.e. using a mean value of −4.98 diopters (D) for the posterior corneal surface (Seitz/Speicher/Savini’s method) [28]. In this event, the preoperative unknown K must still be entered into the Double-K formula and different options are available to estimate it: either an average value is used (e.g. 43.50 D), the preoperative K is obtained by adding the refractive change at the corneal plane to the modified postoperative K value, or it is calculated from the posterior corneal surface parameters [36].

#### Savini’s method and other formulas re-calculating the keratometric index

As explained in the previous section, the keratometric index of 1.3375 used to convert the anterior corneal curvature into diopters is no longer valid after LASIK or PRK. We have shown that this index should be decreased with increasing amount of myopic correction, according to the formula:

Post-refractive surgery index of refraction = 1.338 + 0.0009856*SIRC

Where SIRC = surgical induced refractive change.

Once the keratometric index has been calculated, we can obtain the corneal power using the formula P = (n-1)/R [12]. This method has been proven to give reliable results when combined with the Double-K SRK/T formula [31, 32].

Similar methods have been developed by Camellin and Calossi [11], and Jarade [10], who devised their own formulae to obtain the post-refractive surgery keratometric index (n_post_):

$$ \mathrm{Camellin}:{\mathrm{n}}_{\mathrm{post}}=1.3319+0.00113\times \mathrm{refractive}\ \mathrm{change} $$

$$ \mathrm{Jarade}:{\mathrm{n}}_{\mathrm{post}}=1.3375+0.0014\times \mathrm{refractive}\ \mathrm{change} $$

#### Masket’s formula (for previously myopic and hyperopic eyes)

The IOL power is calculated as if the eye had not undergone previous LASIK or PRK. The IOL power obtained either by Single-K SRK/T (in the case of myopia) or Single-K Hoffer Q (in the case of hyperopia) is then modified according to the formula:

$$ \mathrm{IOL}\ \mathrm{power}\ \mathrm{adjustment}=\mathrm{SIRC}\ast \left(-0.326\right)+0.101 $$

The value thus obtained is added from the standard IOL power calculation in patients with previous myopic laser correction and subtracted in patients with previous hyperopic laser correction [37]. Several studies have shown that this method is quite accurate, although it may give slightly hyperopic results [30–33, 38].

#### Barrett true-K formula

The mathematical formula behind this method has never been published. Current knowledge is that keratometry is modified, the Barrett Universal II formula is used, and a Double-K solution is provided. The refractive change induced by PRK/LASIK is needed for this version of the fomula. However, since it is available for free on the websites of the Asia-Pacific Association of Cataract & Refractive Surgeons (www.apacrs.org) and the American Society of Cataract and Refractive Surgery (www.ascrs.org), some studies have found it leads to accurate refractive results [33, 34]. It is now considered as one of the most reliable options both after myopic and hyperopic PRK/LASIK.

#### Latkany’s regression formula (for previously myopic and hyperopic eyes)

Latkany and colleagues found a linear relationship between the amount of refractive correction and the error in IOL power estimation, as calculated on the basis of conventional keratometry [8]. Depending on which K-value is considered, the regression formula is:

$$ -\left(0.46\times {\mathrm{RX}}_{\mathrm{pre}}+0.21\right)\;\mathrm{for}\ \mathrm{Average}\ \mathrm{K} $$

$$ -\left(0.47\times {\mathrm{RX}}_{\mathrm{pre}}+0.85\right)\;\mathrm{for}\ \mathrm{Flattest}\ \mathrm{K} $$

The value calculated by the regression formula should be added to the IOL power calculated using the corneal power measured by conventional keratometry.

The first equation leads to good results [29, 31].

For eyes with previous hyperopic LASIK, the IOL power should be lowered by adding the result of this regression formula [39]:

$$ -\left(0.27\times \mathrm{pre}\hbox{-} \mathrm{refractive}\ \mathrm{surgery}\ \mathrm{myopic}\ \mathrm{spherical}\ \mathrm{equivalent}+1.53\right) $$

#### Awwad’s formulas

Awwad et al. developed six formulae, depending on which perioperative data are available, to calculate the post-myopic LASIK corneal power [15]. These are derived from the TMS corneal topographer (Tomey, Japan) measurements and can be based either on the Anterior Central Corneal Power at 3 mm (ACCP_3mm_) or simulated keratometry (SimK). When the SIRC is known, the formulae are the following:

$$ \mathrm{Adjusted}\ \mathrm{ACCP}={\mathrm{ACCP}}_{3\mathrm{mm}}-0.16\ast \mathrm{SIRC} $$

$$ \mathrm{Adjusted}\ \mathrm{SimK}=\mathrm{SimK}-0.23\ast \mathrm{SIRC} $$

The accuracy of these formulas has been found to be moderately good [29, 31].

In case of post-hyperopic LASIK eyes [40], the formulae are as follows:

$$ \mathrm{Adjusted}\ \mathrm{ACCP}={\mathrm{ACCP}}_{3\mathrm{mm}}+0.144\ast \mathrm{SIRC} $$

$$ \mathrm{Adjusted}\ \mathrm{SimK}=\mathrm{SimK}+0.165\ast \mathrm{SIRC} $$

The corneal powers obtained by these formulae have to be entered into the Double-K SRK/T (myopic eyes) or Hoffer Q (hyperopic eyes) formulae.

#### Clinical history method

The clinical history method (CHM) was considered the gold standard and the benchmark for comparison for almost twenty years [9, 26, 28, 41, 42]. It was first described by Holladay in 1989 for eyes that had undergone RK and later advocated by Hoffer for eyes with previous LASIK and PRK [43, 44].

The CHM is based on refraction-derived keratometric values and requires knowledge of three perioperative data: preoperative keratometric dioptres (D), surgically-induced refractive change (SIRC) at the corneal plane, and stabilized postoperative refraction.

The postoperative corneal power is obtained by subtracting the SIRC from the preoperative K readings (in the case of hyperopic surgery, the SIRC is added to the preoperative corneal power). The result is given by the formula:

$$ \mathrm{Kpost}=\mathrm{Kpre}-\mathrm{SIRC} $$

Although the results are fairly good when the perioperative data are available (and accurate), refractive surprises have been described, even when the calculated corneal power has been entered into a Double-K formula [4, 5, 8, 26–29, 31]. The CHM also has, in fact, important limitations: not only does it require the presurgical keratometry and the amount of SIRC (information that is often lacking), but it is also highly vulnerable to bad data; its predictive power is seriously affected, for example, by nuclear sclerosis-induced or axial length progression myopia, which can change the post-LASIK/PRK refraction and make the calculation invalid. Therefore, the results are less accurate with respect to other solutions, and is no longer considered the gold standard [29–31, 38].

Other methods requiring historical methods have been described. These include the Diehl-Miller nomogram [45, 46], the Corneal bypass method [47, 48], and the Feiz-Mannis formula and nomogram [7]. Their results have been less satisfactory, especially in eyes with higher amounts of myopic correction [29, 31, 38].

### When preoperative keratometry and refractive change are NOT available

When perioperative data are lacking and clinical charts are not available, other methods can be used. The contact lens method [43, 44], which has been considered the best option for these patients for a long time, has many limitations that preclude its use and is no longer recommended. [49–51] More reliable alternatives are given by the following methods.

#### Shammas-PL and PHL formulas (for previously myopic and hyperopic eyes)

These formulas calculate the corneal power by means of the following equation:

$$ \mathrm{Corneal}\ \mathrm{power}=1.14\ast \mathrm{Kpost}-6.8 $$

where Kpost is the post-refractive surgery keratometry [42].

The calculated corneal power value has to be entered into the original Shammas formula, which does not need the Double-K adjustment as it does not depend on corneal curvature to estimate the ELP (so called Shammas-PL formula) [52]. Several studies reported good results not only in eyes without historical data, but also in those with perioperative data available [29–34, 52]. A specific version (Shammas-PHL formula) can be used for eyes with previous hyperopic LASIK [53]. In this case the formula to obtain the corneal power is as follows:

$$ \mathrm{Corneal}\ \mathrm{power}=1.0457\ast \mathrm{Kpost}-1.9538. $$

#### Maloney’s method

Maloney’s method, as described by Wang and associates in their comparative study [16], is rather similar to the method by Seitz/Speicher/Savini, where a mean value of −4.98 D is used for the posterior corneal power [12]. The main difference lies in the choice of the topographic value, which in Maloney’s method is not the SimK but rather the single power at the center of the axial map. Moreover, in his method, Maloney used a mean power of −4.9D rather than −4.98D.

Hence, corneal power according to Maloney’s method reads as:

$$ \mathrm{K}=\mathrm{measured}\ \mathrm{K}\ast 1.114-4.90 $$

Wang et al. suggested using a modified value of −6.1 D for the posterior corneal power and later further changed it to −5.59 D [16].

#### Haigis-L formula

This formula is available on the IOLMaster (Zeiss, Germany). It is based on the regular Haigis formula, which does not suffer from the above-mentioned formula error, as it does not predict the ELP from the preoperative corneal curvature but from the anterior chamber depth [20, 54]. The Haigis-L formula uses a correlation curve to compensate for the radius and keratometric index errors in the keratometry module of the IOLMaster. The Haigis-L formula consists of two separate corrections for previous myopic and hyperopic excimer laser surgery. The results reported have been good [34, 54].

#### Barrett true-K no history formula

This version of Barrett’s formula has been developed to work without historical data and can be accessed via the same websites reported for the “historical” version (see 2.1.4). The formula has not been published, but the results are good [33].

#### Gaussian optics formula

According to the Gaussian optics formula (GOF), the total corneal power can be calculated from the radius of curvature of both corneal surfaces and the distance between them. The keratometric index is not necessary. The formula is:

$$ \mathrm{P}=\left({\mathrm{n}}_1-{\mathrm{n}}_0\right)/{\mathrm{r}}_1+\left({\mathrm{n}}_2-{\mathrm{n}}_1\right)/{\mathrm{r}}_2-\left(\mathrm{d}/{\mathrm{n}}_1\right)\times \left[\left({\mathrm{n}}_1-{\mathrm{n}}_0\right)/{\mathrm{r}}_1\right]\times \left[\left({\mathrm{n}}_2-{\mathrm{n}}_1\right)/{\mathrm{r}}_2\right] $$

where

n_0_ = refractive index of air (= 1.000)

n_1_ = refractive index of cornea (= 1.376)

n_2_ = refractive index of aqueous humor (= 1.336)

r_1_ = radius of curvature of anterior corneal surface (in meters)

r_2_ = radius of curvature of posterior corneal surface (in meters)

d = corneal thickness in meters

A Scheimpflug camera or an anterior segment OCT are necessary to measure the posterior corneal curvature. Studies using these devices have shown that in unoperated eyes, the GOF provides a corneal power that is lower by 1.2 D with respect to standard keratometry [55–57]. This difference makes constant optimization mandatory if corneal power by the GOF has to be entered into IOL power calculation formulas. Once complete, the results are accurate and close to those obtained with simulated keratometry in unoperated eyes [56]. Theoretically, the same results should be achieved in post-LASIK eyes if a Double-K formula or the Haigis formula are used, but this has not been proven. To overcome the systematic difference between the corneal power calculated by the GOF and by the standard keratometric index, Borasio et al. modified the GOF and developed the BESSt formula. Good results have been reported by the authors in their original manuscript, but no further studies have validated it [58].

#### Corneal ray-tracing

Instruments measuring the curvature of both corneal surfaces can calculate corneal power by means of ray-tracing based on Snell’s law. For each point on the corneal map, the angle of incidence is calculated relative to the anterior surface normal for incoming parallel rays. The angle of refraction is calculated using Snell’s law. This angle of refraction is used to determine the nonparallel direction of incoming rays relative to the posterior surface normal and to calculate the angle of incidence for the posterior surface. A new angle of refraction is calculated for the posterior surface using Snell’s law. This final angle of refraction is used to calculate the intersection of the ray along the (0,0) axis, and the resultant focal length is used to determine the corneal power for that point on the map. After LASIK and PRK, the corneal power calculated by this method has to be entered into Double-K formulas. Since in unoperated eyes corneal power by ray-tracing is lower than the corresponding value calculated with the keratometric index by about 0.5 to 0.7 D, constant optimization is required. [56, 59, 60] The Galilei (Ziemer Opthalmic Systems AG, Port, Switzerland), a dual Scheimpflug analyzer combined with a Placido disc, offers the Total Corneal Power; the Pentacam, a rotating Scheimpflug camera, offers the Total Corneal Refractive Power; the Sirius (CSO, Firenze, Italy), a rotating Scheimpflug camera combined with a Placido disc corneal topographer, offers the Mean Pupil Power. In unoperated eyes, these values have been shown to lead to accurate refractive outcomes when entered into third-generation IOL power formulas [56, 59, 60]. Data in post-refractive surgery eyes are still lacking.

#### IOL power calculation by ray-tracing

Ray-tracing can also be used to calculate the IOL power and thus replace standard theoretical thin lens formulas. The refraction of rays at each optical surface from the tear film to the retina is calculated using Snell’s law. After corneal refractive surgery, ray-tracing offers the great advantage of not being subject to these three errors [1]: i) the keratometric index problem, since ray-tracing does not rely on the keratometric index, but is based on real curvature data from both corneal surfaces, ii) the “radius problem”, since ray-tracing can be calculated over any corneal diameter, and iii) the formula error, since the IOL position can be estimated without relying on the anterior corneal curvature (moreover, all ray-tracing methods do not estimate the ELP, which is a theoretical value corresponding to secondary principal plane of the IOL and cannot be measured, but the real geometric position of the IOL). Finally, ray-tracing does not require historical data and can include corneal aberrations in IOL power calculation [61]. Several studies have shown encouraging results with this approach in eyes with previous LASIK or PRK [62–65].

Different solutions are commercially available. They include Okulix and Phaco-Optics [66, 67], which support exact ray-tracing and can be applied to measurements from most devices. Another interesting alternative is the internal software of the Sirius instrument. Each software has its own IOL position prediction algorithm.

A similar solution is provided by the RTVue spectral-domain OCT, which calculates the corneal power by means of the GOF and uses a vergence formula to calculate the IOL power. The vergence formula is similar to ray-tracing, although it is valid only in the paraxial limit and the IOL is considered as a thin lens [68]. The ELP is predicted by a regression formula based on preoperative measurements of ACD, lens thickness and axial length. In case of previous LASIK or PRK, the OCT net corneal power calculated by the GOF is converted into an effective corneal power (ECP), which is different in cases of myopic or hyperopic surgery. The results have been promising [68–70].

#### Aphakic refraction technique

The aphakic refraction technique was developed by Ianchulev [71] and later adapted by Mackool [72]. In this technique, phacoemulsification is performed without inserting the IOL. Subsequently, Ianchulev used a portable autorefractor to assess the aphakic refraction, while the patient was still reclined on the operating table. Conversely, Mackool suggested waiting thirty minutes and then taking the patient to an examining room and measuring the aphakic refraction; finally the patient came back to the operating room and the chosen IOL was implanted, a method few have utilized.

The formulae for IOL power calculation are slightly different:

$$ \mathrm{IOL}\ \mathrm{power}=\mathrm{aphakic}\ \mathrm{refraction}\ \left(\mathrm{spherical}\ \mathrm{equivalent}\right)\times \kern0.37em 2.01449\ \left(\mathrm{Ianchulev}\right) $$

$$ \mathrm{IOL}\ \mathrm{power}=\mathrm{aphakic}\ \mathrm{refraction}\ \left(\mathrm{spherical}\ \mathrm{equivalent}\right)\times \kern0.37em 1.75\ \left(\mathrm{Mackool}\right) $$

The results reported by both authors in small groups of patients were excellent.

Based on the aphakic refraction technique, a specific instrument (Ora System, Alcon, Fort Worth, TX) has been designed to calculate the IOL power in the operating room once the crystalline lens has been extracted. The Ora System is composed of an optical head that attaches directly to the surgical microscope and is connected to a computer processor. When the patient is on the table, it measures intraoperative wavefront aberrometry and enables real-time IOL power calculations. Good results have been recently reported in two series of post-LASIK and post-PRK eyes [72, 73].

## Which results can be expected and which methods are more accurate?

The many studies that compared the above-mentioned methods showed that all are quite accurate, the only exception being the CHM. [30–34, 70] In most cases, between 60 and 70% of eyes can be expected to have an absolute prediction error (PE) within ±0.5 (D); when historical data are lacking, the percentage is slightly lower i.e. between 50 and 60%, but increases up to 70% when ray-tracing or intraoperative aberrometry are used. [34, 64, 70] These percentages are close to those achieved in unoperated eyes [74]. However, we believe it is mandatory to advise patients that perfection i.e. 100% of eyes with a PE < 0.50 D, is not possible, as they may be accustomed to the outcomes of excimer laser surgery, which are considerably more accurate than those obtained with IOL power calculation. Tables 1 and 2 show the results reported by previously published papers, respectively, in eyes with and without historical data.

**Table 1:** Refractive outcomes of IOL power calculation in eyes with historical data

	Historical data		N	MedAE (D)	Mean Error (D)*		% < 0.5 D
	K	Rx			Mean ± SD	Range	
Awwad [32]	Yes	Yes	30	0.36	−0.38 ± 0.51	−1.27, 0.85	56.7
Barrett True-K [33]	No	Yes	58	0.33	−0.01 ± 0.55	−0.96, 1.53	67.2
Barrett True-K [34]	No	Yes	28	0.33	0.06 ± 0.98	−2.02, 2.61	67.9
Latkany [32]	Yes	Yes	30	0.48	−0.33 ± 0.54	−1.22, 0.73	56.7
Masket [32]	Yes	Yes	30	0.34	0.22 ± 0.44	−0.67, 1.11	73.3
Masket [33]	No	Yes	58	0.32	0.29 ± 0.79	−1.26, 1.59	60.3
Masket [34]	No	Yes	28	0.32	0.21 ± 1.07	−1.30, 3.61	64.3
Masket [30]	Yes	Yes	170	–	0.29 ± 0.79	−3.55, 4.59	55.3
Savini [32]	Yes	Yes	30	0.29	−0.01 ± 0.48	−1.03, 1.00	70.0
Seitz/Speicher [32]	Yes	Yes	30	0.42	0.14 ± 0.48	−0.94, 0.99	60.0

MedAE = median absolute error in refraction prediction; SD = standard deviation; D = diopters

*A negative number shows a myopic outcome

**Table 2:** Refractive outcomes of IOL power calculation in eyes with no historical data

	N	MedAE (D)	Mean Error (D)*		% < 0.5 D
			Mean ± SD	Range	
Barrett True-K No-Hx [33]	30	0.41	−0.20 ± 0.64	−1.55, 1.28	63.3
Barrett True-K No-Hx [34]	104	0.42	−0.07 ± 0.89	−1.86, 2.65	58.7
Haigis-L [30]	170	–	−0.26 ± 1.13	−3.40, 6.34	40.2
Haigis-L [33]	58	0.58	−0.34 ± 0.74	−1.65, 2.86	48.3
Haigis-L [33]	30	0.62	−0.50 ± 0.65	−1.94, 1.03	46.7
Haigis-L [34]	104	0.39	−0.07 ± 0.88	−2.15, 2.04	55.8
Haigis-L [70]	39	0.26	–	–	69.0
OCT [34]	104	0.35	−0.20 ± 0.73	−2.10, 1.33	68.3
OCT [70]	39	0.28	–	–	72.0
Intraoperative aberrometry [70]	39	0.29	–	–	74.0
Ray-tracing [64]	21	0.25	0.13 ± 0.49	−0.85, 1.28	71.4
Shammas No-Hx [30]	170	–	−0.10 ± 1.02	−5.42, 6.16	53.8
Shammas No-Hx [30]	30	0.53	−0.34 ± 0.72	−1.75, 1.46	50.0
Shammas No-Hx [34]	104	0.48	−0.34 ± 0.94	−2.09, 2.99	52.9

MedAE = median absolute error in refraction prediction; SD = standard deviation; D = diopters

*A negative number shows a myopic outcome

## Methods developed to calculate IOL power after RK

A smaller number of methods are available to calculate the corneal power when phacoemulsification and IOL implantation are performed in eyes that previously underwent RK. If the preoperative data and the postoperative refraction are known, the CHM has long been considered the best option, although its reliability may be limited by post-RK hyperopic shift.

Using standard keratometric values leads to corneal power underestimation (and consequent myopic error after IOL implantation) because the keratometric index (1.3375) is altered in the opposite direction compared to myopic PRK and LASIK [22]. The corneal power underestimation may be compensated by the radius error caused by the small optical zone. However, in order to reduce the radius error, it is recommended to discard SimK values and consider more central measurements of the corneal power, such as the ACCP_3mm_ of the TMS or the Effective Refractive Power (EffRP) given in the Holladay Diagnostic Summary of the EyeSis Corneal Analysis System [75–77]. These values should be entered into Double-K formulas to avoid an erroneous ELP prediction. Packer and coauthors reported excellent results by inserting the EffRP in the Holladay 2 formula [76]. Awwad et al. achieved similar results entering the ACCP_3mm_ from the TMS corneal topography into the Double-K Holladay 1 formula [78]. Alternatively, the keratometry readings of the IOLMaster (Carl Zeiss, Jena, Germany), which are taken along a relatively small diameter (2.5 mm), may be entered into the Haigis formula, which is not affected with the ELP prediction error. With this method, Geggel achieved good results [79].

In order to solve both the keratometric and radius errors, the corneal power may be calculated by ray-tracing. So far, however, there are no published studies to confirm this hypothesis.

When dealing with post-RK eyes and evaluating the refractive outcome of IOL power calculation, surgeons should also take into account the diurnal variation of refraction.

## Conclusions

Understanding the reasons leading to refractive errors in these eyes is highly recommended and helps avoiding errors in many cases. For this reason we hope that any cataract surgeons will become familiar with the keratometric index error, the radius error and the ELP error.

When selecting the IOL power, we suggest to look at the results of the most accurate formulas (according to the published studies) for any specific patient, relying on formulas for eyes with clinical data, when these are available, and formulas for eyes without clinical data, when these cannot be retrieved.

Patients should always be advised that a prediction error in refraction can occur in these cases, even with the most advanced technologies.
